# Chronic diuretic therapy attenuates renal BOLD magnetic resonance response to an acute furosemide stimulus

**DOI:** 10.1186/1532-429X-16-17

**Published:** 2014-02-03

**Authors:** Michael E Hall, Michael V Rocco, Timothy M Morgan, Craig A Hamilton, Matthew S Edwards, Jennifer H Jordan, Justin B Hurie, W Gregory Hundley

**Affiliations:** 1 Department of Medicine, Division of Cardiology, University of Mississippi Medical Center, Jackson, Mississippi, USA; 2 Department of Medicine, Section on Nephrology, Wake Forest University School of Medicine, Winston-Salem, USA; 3 Department of Biostatistical Sciences, Wake Forest University School of Medicine, Winston-Salem, USA; 4 Department of Biomedical Engineering, Wake Forest University School of Medicine, Winston-Salem, USA; 5 Department of Vascular Surgery, Wake Forest University School of Medicine, Winston-Salem, USA; 6 Department of Medicine, Section on Cardiology, Wake Forest University School of Medicine, Winston-Salem, USA; 7 Department of Radiology, Wake Forest University School of Medicine, Winston-Salem, USA; 8 Wake Forest School of Medicine, Medical Center Boulevard, Winston-Salem, NC 27157-1045, USA

**Keywords:** Renal BOLD, Oxygenation, Furosemide, Renal artery stenosis

## Abstract

**Background:**

Blood Oxygen Level Dependent (BOLD) magnetic resonance (MR) is a novel imaging tool that detects changes in tissue oxygenation. Increases in renal oxygenation in response to a standard 20 mg intravenous furosemide stimulus have been evaluated to assess kidney viability in patients with renal artery stenosis (RAS). The effect of prior exposure to furosemide on the ability of BOLD MR techniques to evaluate renal function is unknown.

This study tested the hypothesis that chronic loop diuretic therapy is associated with attenuated responses in renal tissue oxygenation as measured by BOLD MR with an acute 20 mg intravenous furosemide stimulus in participants undergoing evaluation for RAS.

**Methods:**

Thirty-eight participants referred for evaluation of RAS were recruited for this study. We examined renal cortical and medullary BOLD signal (T2*) intensities before and after a 20 mg intravenous furosemide stimulus. Additionally, we measured changes in renal artery blood flow using phase contrast techniques.

**Results:**

After controlling for covariates age, race, gender, diabetes, glomerular filtration rate, body mass index, and stenosis severity, daily oral furosemide dose was an independent, negative predictor of renal medullary T2* response (p = 0.01) to a standard 20 mg intravenous furosemide stimulus. Stenosis severity and ethnicity were also significant independent predictors of changes in T2* signal intensity in response to an acute furosemide challenge. Changes in renal blood flow in response to acute furosemide administration were correlated with changes in T2* in the renal cortex (r = 0.29, p = 0.03) but not the medulla suggesting changes in renal medullary oxygenation were not due to reduced renal medullary blood flow.

**Conclusions:**

Chronic furosemide therapy attenuates BOLD MR responses to an acute furosemide stimulus in patients with RAS being evaluated for renal artery revascularization procedures. Thus, patients who are chronically administered loop diuretics may need a different dosing strategy to accurately detect changes in renal oxygenation with BOLD MR in response to a furosemide stimulus.

## Background

Improvement in renal tissue oxygenation manifested by increased Blood Oxygen Level Dependent (BOLD) magnetic resonance (MR) signal intensity after intravenous (IV) furosemide has recently been identified as a potential noninvasive marker of “renal viability” that may indicate kidneys most suitable for renal artery revascularization procedures [1,2]. In these MR procedures, BOLD images are generally acquired prior to and fifteen minutes after a standard IV 20 mg dose of furosemide [1-3]. If a kidney subtended by a renal arterial luminal narrowing ≥70% is unable to increase tissue oxygenation in response to an IV 20 mg furosemide stimulus, it may be less likely to significantly improve its function after renal artery revascularization (as evidenced by reduction in systemic arterial pressure and increased glomerular filtration rate [GFR]).

Importantly however, the potential influence of chronic oral furosemide treatment on the response to an acute furosemide stimulus during the BOLD MR imaging procedure is unknown. This point is important because patients with severe renal artery stenosis (RAS) receiving chronic loop diuretic therapy may have an attenuated BOLD MR response to acute furosemide administration. If this is the case, an attenuated BOLD MR response to furosemide could incorrectly be interpreted as evidence that the patient might not benefit from renal artery revascularization procedures when in fact they may be a suitable candidate. In this study, we prospectively evaluated renal cortical and medullary BOLD MR measures before and after 20 mg of IV furosemide in patients aged ≥ 55 years who were referred for RAS evaluation. We examined the BOLD MR responses to 20 mg of IV furosemide after accounting for the chronic administration of oral loop diuretic therapy.

## Methods

### Study population

This study was approved by the Institutional Review Board (IRB) at the Wake Forest School of Medicine and all study participants provided written, witnessed informed consent. We included 38 participants referred for evaluation of RAS based on existing clinical suspicion or findings from existing non-invasive imaging studies including renal Doppler examinations, contrast angiography or CT. Participants were ineligible for enrollment if they exhibited a contraindication for MR (implanted metal, pacemakers, defibrillators, other electronic devices or claustrophobia), active acute coronary or cerebral or peripheral arterial symptoms, severe aortic stenosis or other significant valvular disease, or those with a contraindication to the receipt of furosemide. After enrollment, GFR was estimated in each participant using the Chronic Kidney Disease Epidemiology Collaboration (CKD-EPI) equation [4] using the serum creatinine value obtained from a blood draw obtained within four weeks prior to each MR examination. Participants were asked to abstain from taking any diuretics including furosemide at least 12 hours prior to the MR scan. The records and clinical characteristics of each participant were reviewed in accordance with Wake Forest School of Medicine IRB policies.

### MR Imaging protocol

Comprehensive MR imaging examinations were performed on a Magnetom Avanto 1.5 Tesla scanner (Siemens Medical Solutions USA, Malvern, Pennsylvania) using a phased-array surface coil applied across the abdomen to optimize signal to noise. Electrocardiographic (ECG) leads and respiratory gating bellows were applied to account for cardiac and respiratory motion respectively. Blood pressure and heart rate were monitored periodically during the MR examination to ensure hemodynamic stability.

A three-dimensional (3D), segmented steady-state free-precession sequence with non-selective radiofrequency excitation was utilized to acquire non-contrasted angiograms of the renal arteries. The fields of view (FOV) ranged from 30-40 cm to cover the entire abdomen in the axial position to obtain 3D volume acquisitions. Imaging parameters included a repetition time (TR) of 2.3 ms, an echo time (TE) of 1 ms, a 90° flip angle, a readout bandwidth of 980 Hz per pixel, a 256 × 256 matrix, and a total of approximately 40-50 3D partitions with a slice thickness of 3 mm. A parallel imaging technique, generalized autocalibrating partially parallel acquisition (GRAPPA) with an acceleration factor of 2 was applied to shorten scan times. Percentage stenosis of each renal artery was visually estimated by a blinded board-certified cardiologist and cardiovascular imager.

After locating the renal arteries, an image series of the vessels in double-obliqued cross-sectional orientation was obtained to ensure a circular lumen throughout the cardiac cycle to minimize partial volume effects during image acquisition. Interleaved, phase-contrast gradient-echo sequences were used to determine cardiac cycle-dependent measurements of vessel area and velocity according to previously published techniques from our laboratory [5]. These sequences were positioned in an oblique plane 2 cm distal to the origin of the renal artery at the aorta. These scans utilized 7 mm thick slices with a 256 × 256 matrix, 32 cm FOV (yielding voxel sizes of 0.94 × 0.94 × 7 mm for the renal artery), a 40° flip angle, a TR of 11 ms, and a TE of 3.5 ms.

During suspended respiration, three-plane, single-shot, fast spin-echo localizing images of the kidneys were performed followed by additional scout images oriented parallel to the coronal axis of each kidney [2]. BOLD imaging consisted of a 2-D fast spoiled gradient echo sequence with eight echoes (TE’s ranged from 2.5-30 ms) obtained at each slice location. BOLD imaging parameters included a slice thickness of 10 mm, a 224 × 160-192 imaging matrix, a 32-40 cm FOV, a 45° flip angle, and a TR of 140 ms [6]. The FOV was adjusted based on participants’ body sizes and the imaging matrix and TR’s were adjusted in participants with diminished breath hold capabilities.

BOLD images were acquired during suspended respiration with coronal slices through the middle of each kidney. Signal intensity versus TE data on a voxel by voxel basis were used to generate parametric images of T2*. After completion of the initial BOLD acquisitions, each participant received 20 mg IV furosemide according to previously published methods [1,2]. Fifteen minutes after receipt of furosemide, the renal arterial flow (phase contrast) and T2* (BOLD) acquisitions were repeated.

After acquisition, images were transferred to a postprocessing station for analysis. A single person blinded to clinical participant characteristics performed all image analyses. Images (either BOLD or phase contrast) were excluded if there was severe motion artifact, bowel gas artifacts or if image quality was degraded and renal segments (cortex versus medulla) or renal artery were not able to be clearly identified. Phase contrast and BOLD images were analyzed using post-processing in custom-written MATLAB (The Mathworks, Inc.; Natick, Massachusetts). The cross-sectional area of the renal arterial lumen was defined on the magnitude image of the reference scan by a region of interest (ROI) which was then superimposed on the velocity map for each corresponding frame of the cardiac cycle and the mean velocities were obtained by measuring the average pixel intensity within the ROI.

Renal cortical and medullary T2* values were determined by manually tracing ROI’s on arterial spin labeling images with the highest corticomedullary differentiation. Each ROI contained at least 5 voxels. These ROI’s were then copied onto the corresponding parametric T2* maps (both pre-and post-furosemide). Care was taken to ensure that the ROI was drawn in clearly identifiable cortical or medullary segments. Areas of artifact or cysts were avoided. These same ROI’s were then copied onto the post-furosemide images to ensure similar segmental position within each kidney.

### Statistical analysis

All statistical analyses were performed using SAS 9.2 software (SAS Institute, Cary, North Carolina). Subjects were classified into two groups: chronic furosemide therapy versus furosemide naïve. The dosage of chronic furosemide therapy was also considered as a continuous covariate. Descriptive statistics were performed on participants on chronic furosemide therapy and compared to furosemide naïve participants. A p-value of < 0.05 was considered to be statistically significant. Univariate, multivariable, and backwards stepwise regression analyses using mixed model procedures, with kidney side and kidney region considered as repeated measures, were performed to evaluate the effect of kidney region, age, gender, ethnicity, estimated GFR, stenosis severity, presence of diabetes, body mass index, use of ACEI/ARBs, number of antihypertensive medications, and chronic daily furosemide dose (total daily dose in mg) on changes in renal tissue oxygenation as measured by percentage change in T2* signal intensities before and after 20 mg IV furosemide administration. Similar statistical models were used to evaluate the effects of these variables on percentage changes in main RBF with kidney side as the only repeated measure. Percentage change in RBF was determined using the change in the measured flow from the pre-furosemide image to the post-furosemide image divided by the pre-furosemide (baseline) measurement. The correlation between percentage changes in RBF using phase contrast and percentage change in renal tissue oxygenation (T2*) was estimated using Pearson’s correlation coefficient separately within each kidney region.

## Results

A total of 38 participants were evaluated in this study. Descriptive statistics of the participants, subdivided by the concurrent use of chronic furosemide therapy are displayed in Table 1. Participants receiving chronic furosemide therapy exhibited lower GFR’s (p = 0.005), required more antihypertensive medications (p = 0.0001), were more likely to be diabetic (p = 0.01), and were less commonly treated with angiotensin converting enzyme (ACE) inhibitors or angiotensin receptor blockers (ARBs, p = 0.006). Among the 15 participants chronically administered furosemide, the total daily dose range ranged from 20 mg daily to 160 mg daily.

**Table 1:** Participant characteristics

**Participant characteristics**	**Furosemide naïve**	**Chronic furosemide**	**p-value**
	**(n = 23)**	**(n = 15)**	
Age (years)	68 ± 8	69 ± 10	0.49
Race			0.28
White (%)	78	67	
African American (%)	22	33	
Gender (% male)	35	40	0.65
Body mass index (kg/m^2^)	29.4 ± 6.5	30.3 ± 7.1	0.56
GFR (mL/min/1.73 m^2^)	60.1 ± 31.6	44.6 ± 14.7	0.005*
Renal artery stenosis (mean,%)			
Left	26	24	0.70
Right	26	21	0.82
Renal blood flow (mean, ml/min)			
Left	187	127	0.17
Right	231	196	0.50
Chronic kidney disease stage (n)			0.01*
Stage 1 (GFR > 90)	5 (22%)	0 (0%)	
Stage 2 (GFR 60-89)	5 (22%)	2 (13%)	
Stage 3 (GFR 30-59)	9 (39%)	9 (60%)	
Stage 4 (GFR 15-29)	4 (17%)	4 (27%)	
Stage 5 (GFR < 15)	0 (0%)	0 (0%)	
Hypertension diagnosis (%)	91	100	0.25
Number of antihypertensive meds (n)	2.7 ± 1.6	4.3 ± 1.0	0.0001*
Hemoglobin (g/dL)	12.7 ± 2.3	12.1 ± 1.8	0.25
Diabetes diagnosis (%)	30	60	0.01*
Patients on statin therapy (%)	65	73	0.45
Patients on ACEI’s or ARB’s (%)	65	33	0.006*

Mean ± SE are displayed. *GFR* glomerular filtration rate, *ACEI* angiotensin converting enzyme inhibitors, *ARB* angiotensin receptor blockers. *represents p<0.05.

### Effect of chronic furosemide therapy on T2* response to acute furosemide administration

**Figure 1:**
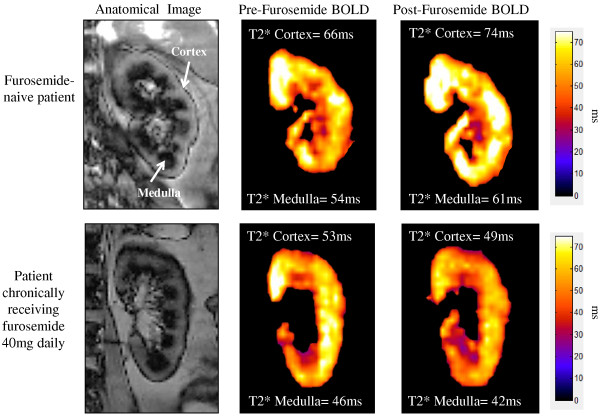
**MR images of the left kidneys of two participants: furosemide naïve (top row) and chronic furosemide administration of 40 mg/day (bottom row).** The anatomic images in the panel in the left column are generated from a non-contrast arterial spin labeling technique in which the renal cortical segments appear gray and the medullary segments appear dark (white arrows). Corresponding T2* maps representing tissue oxygenation (Blood Oxygen Level Dependent or BOLD) before (middle column) and after (right column) a 20 mg intravenous furosemide stimulus. On these images the color scale intensity represents the T2* value for that corresponding voxel. As shown, the furosemide naïve participant increased both cortical and medullary T2* signal intensities in response to a 20 mg intravenous furosemide stimulus by 12% and 13% respectively. However, the cortical and medullary T2* percent change in signal intensity decreased (bottom right panel) by 7% and 9% respectively after a 20 mg intravenous furosemide stimulus.

**Figure 2:**
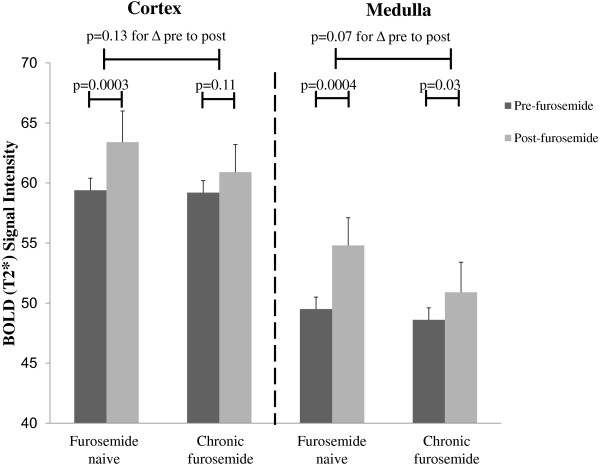
Bar graph displaying mean ± SE renal cortical and medullary BOLD signal intensities (T2*) prior to (light gray) and after (black) a 20 mg intravenous furosemide stimulus in furosemide naïve participants compared with participants chronically receiving furosemide.

Representative MR scans of participants who were or were not receiving chronic furosemide therapy are shown in Figure 1. Overall, furosemide naïve participants experienced significant increases in cortical T2* values after the 20 mg IV furosemide stimulus whereas those receiving chronic furosemide did not (Figure 2). Both furosemide naïve and recipients of chronic oral furosemide experienced an increase in renal medullary T2* signal intensities after the 20 mg IV furosemide stimulus albeit with a strong trend toward attenuation of the response in those chronically prescribed oral furosemide (p = 0.07).

Using repeated measures multiple regression analyses, change in total kidney oxygenation (T2*) was univariately predicted by kidney region (cortex versus medulla, β = - 3.76 ± 1.81, p = 0.04) and chronic daily furosemide dose (β = - 0.06 ± 0.03, p = 0.04). Multivariable analyses demonstrated significant negative effects for both region (β = - 4.06 ± 1.89, p = 0.04) and chronic furosemide dose (β = - 0.08 ± 0.03, p = 0.02) after accounting for age, GFR, gender, BMI, percentage stenosis of the renal artery, diagnosis of diabetes, use of ACE inhibitors/ARBs, and number of antihypertensive medications. There were no significant interaction effects between the variables kidney region (cortex vs medulla) and total daily furosemide dose or GFR and furosemide dose.

There was a significant difference in the GFR’s of the two groups (p = 0.01). Therefore, we performed analyses after excluding the five participants with high normal renal function (GFR ≥ 90 mL/min/1.73 m^2^) in order to make the groups more comparable. Kidney region (cortex vs medulla, β = -4.23 ± 1.96, p = 0.04) and total daily furosemide dose (β = -0.06 ± 0.03, p = 0.04) remained as significant predictors of attenuated T2* signal intensity changes in response to an acute furosemide stimulus.

**Figure 3:**
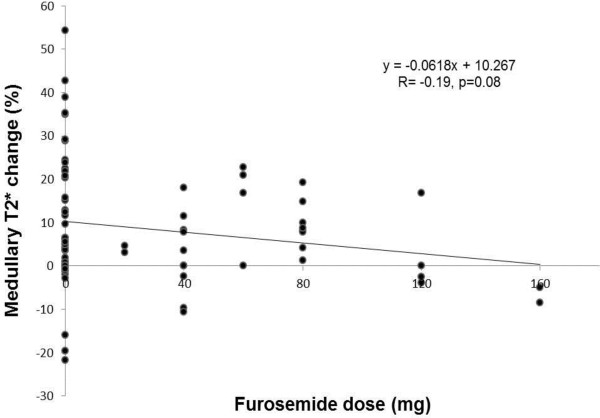
Scatter plot demonstrating the relationship between daily oral furosemide dose (x-axis) and the percentage change in medullary T2* values after 20 mg intravenous furosemide (y-axis). The circular symbols (●) represent data from a single participant. The regression equation and line are displayed. The asterisk (*) represents p <0.05.

Due to the significant effect of kidney region on T2* changes in response to acute furosemide administration, we also evaluated covariates within each region of the kidney. Univariate and backwards stepwise regression analyses were performed (Table 2). Using backwards stepwise regression, three variables (ethnicity [p = 0.004], stenosis severity [p = 0.04, and chronic daily furosemide dose [p = 0.01]) were significant independent predictors of changes in T2* signal intensity in response to an acute furosemide stimulus. The results of our model indicate that a patient receiving 40 mg of furosemide twice daily or 80 mg once daily would be predicted to exhibit a 9% attenuation of the T2* signal intensity response in the renal medulla to a 20 mg IV furosemide stimulus, assuming no differences in the other variables. The association between receipt of higher chronic daily doses of furosemide and attenuated renal medullary T2* signal intensity changes in response to a 20 mg IV furosemide stimulus nearly reached statistical significance (r = -0.19, p = 0.08, Figure 3).

**Table 2:** Univariate and backwards stepwise regression analyses of percentage change in renal medullary tissue oxygenation (T2* signal intensity) in response to a 20 mg intravenous furosemide stimulus

**Variables**	**Univariate**		**Backwards stepwise regression**	
	**Parameter estimate (SE)**	**p-value**	**Parameter estimate (SE)**	**p-value**
Age	0.32 (0.29)	0.28		
Glomerular filtration rate	0.05 (0.11)	0.65		
Gender	2.27 (4.31)	0.61		
Ethnicity	-14.4 (4.61)	0.006*	-13.1 (4.09)	0.004*
Stenosis severity (%)	-0.12 (0.06)	0.04*	-0.11 (0.05)	0.04*
Diabetes diagnosis	0.72 (4.61)	0.88		
Body mass index	-0.49 (0.33)	0.15		
Chronic daily furosemide dose (mg/day)	-0.09 (0.05)	0.09	-0.11 (0.04)	0.01*
ACEI/ARB use	6.41 (4.64)	0.18		
Antihypertensive medications (number)	0.98 (1.74)	0.58		

### Renal artery blood flow response to acute furosemide

**Figure 4:**
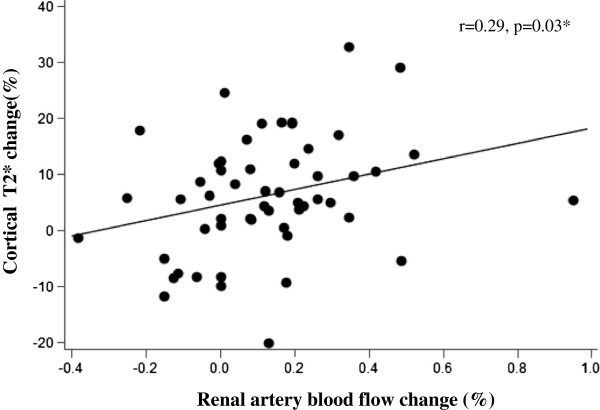
Scatter plot demonstrating the relationship between percentage change in renal artery blood flow and percentage change in renal cortical T2* values (x-axis) after 20 mg intravenous furosemide (y-axis). The regression line, Pearson’s correlation coefficient, and p-value are displayed.

Renal artery blood flow (RBF) was measured before and after acute 20 mg IV furosemide administration using phase contrast techniques in the main renal artery. Baseline RBF was lower, although not statistically significant, in participants chronically administered oral furosemide compared to furosemide naïve participants (160 ± 14 ml/min vs. 210 ± 27 ml/min, p = 0.11). This reduction in baseline RBF was independent of stenosis severity as there was no difference in stenosis severity between the two groups (p = 0.69). RBF increased to 238 ± 28 ml/min (p = 0.005) in furosemide naïve participants. RBF did not significantly increase from pre to post-furosemide measures in participants chronically receiving daily oral doses of furosemide (165 ± 16 ml/min, p = 0.37). Changes in RBF were moderately correlated with changes in cortical T2* values (Figure 4, r = 0.29, p = 0.03). There was no significant correlation between changes in RBF and changes in renal medullary T2* (r = 0.04, p = 0.74).

## Discussion

The results of this study indicate that chronic oral furosemide administration attenuates the increase in renal medullary oxygenation in response to an acute 20 mg IV furosemide stimulus measured by BOLD MR, that higher daily doses of furosemide are associated with reduced increases in the medullary BOLD responses to a standard furosemide challenge, and that changes in RBF are correlated with changes in renal tissue oxygenation in the cortex but not in the medulla.

Renal artery stenosis is a complex pathophysiologic process that can result in malignant hypertension (HTN), renal dysfunction and exacerbation of congestive heart failure [7-9]. Renal artery revascularization procedures, either surgical or percutaneous, have been utilized to treat renovascular HTN in patients with malignant HTN, declining renal function, or those who develop pulmonary edema based on current guideline recommendations [10]. However, less than half of patients who undergo these revascularization procedures actually receive significant clinical benefit defined as improvement in renal function and GFR, or blood pressure [7]. To date, ideal strategies to identify patients who will derive a benefit from renal artery revascularization procedures remain elusive.

Recently, BOLD MR imaging has been evaluated as a potential tool to determine if a kidney with a renal artery lesion can increase oxygenation in response to a 20 mg IV furosemide stimulus [1,2]. Small cross-sectional studies suggest that a kidney with the ability to improve tissue oxygenation (manifested by an increase in BOLD signal intensity) in response to a furosemide stimulus may be more likely to benefit from a revascularization procedure [1,2,11]. Furosemide, a loop diuretic, blocks transport of the sodium-potassium-2chloride (Na-K-2Cl) transporter located in the thick ascending limb of the loop of Henle (TALH) within the renal medulla [12]. Luminal Na + uptake via the apical membrane Na-K-2Cl transporter is coupled to basolateral membrane transport which depends on Na-K-ATPase activity, an oxygen (O_2_)-dependent process [13,14]. Therefore, blocking the Na-K-2Cl transporter reduces renal medullary O_2_ consumption and increases tissue oxygenation.

Since many patients with severe renovascular disease require loop diuretics to prevent hypervolemia, it is important to determine the effect of chronic loop diuretic use on the BOLD MR response to an acute stimulus with furosemide. Otherwise, patients receiving chronic loop diuretic therapy who demonstrate attenuated increases in T2* signal intensities after acute furosemide administration may be inappropriately deemed less likely to receive clinical benefit from renal artery revascularization procedures. Our results demonstrate that chronic oral furosemide consumption is associated with attenuated increases in medullary oxygenation after 20 mg of IV furosemide (Figure 2) despite abstaining from furosemide ingestion greater than 12 hours prior to BOLD MR examination. The half-life of furosemide is 30 to 60 minutes, therefore 12 hours should be more than adequate time to withhold furosemide. The results of our model indicate that a patient receiving 40 mg of furosemide twice daily or 80 mg once daily would be predicted to exhibit nearly a 10% attenuation of the T2* signal intensity response in the renal medulla to a 20 mg IV furosemide stimulus, assuming no differences in the other variables.

Despite worsened renal dysfunction and a higher prevalence of diabetes in the chronic furosemide group, pre-furosemide cortical and medullary T2* values were similar to furosemide naïve participants. This observation illustrates the point that unless patients have a critical renal arterial narrowing, the renal cortex and medulla are able to maintain tissue oxygenation, at least at rest. However, upon a 20 mg IV furosemide challenge, participants receiving chronic furosemide did not significantly increase T2* signal intensities in the cortex. Although these participants did exhibit a significant increase in T2* signal intensities after 20 mg IV furosemide in the renal medulla compared to pre-furosemide values, they exhibited attenuated increases in medullary T2* values compared to furosemide naïve participants (Figure 4).

After adjustment for covariates age, gender, race, GFR, diabetes, stenosis severity and BMI, number of antihypertensive medications, use of ACE inhibitors or ARBs, chronic daily furosemide dose was a significant negative predictor of the renal medullary T2* response to an acute furosemide stimulus. Although it did not quite meet statistical significance (p = 0.07) due to a relatively small number of participants in this study, total daily furosemide dose was associated with attenuated T2* signal intensity changes in response to an acute furosemide stimulus. However, chronic daily furosemide dose did not predict the response in the cortex. This finding is consistent with the site of action of loop diuretics such as furosemide which act on the TALH [15]. In rodents, furosemide significantly increased O_2_ levels measured by O_2_-sensitive electrodes in the renal medulla [16]. This increase in medullary oxygenation was due to decreased tubular O_2_ consumption as medullary blood flow was decreased by nearly 30%.

White race was also associated with significantly attenuated T2* responses in the medulla to an acute furosemide stimulus. Textor *et al*. found higher baseline BOLD MR renal medullary R2* values (inverse of T2*) in African Americans, compared to whites [17]. After a furosemide stimulus, however, these R2* levels decreased to the same values as in whites. These findings suggest increased O_2_ consumption and TALH Na + reabsorption in African Americans compared to whites. If whites reabsorb less Na + in the TALH, they would be expected to have less inhibition of the Na-K-2Cl transporter in response to furosemide. Therefore, our findings corroborate those of Textor *et al*. suggesting that African Americans have increased Na + reabsorption in the TALH compared to whites. The mechanisms involved in the augmented TALH Na + reabsorption in African Americans are poorly understood and warrant further study.

Higher chronic furosemide doses were associated, albeit weakly, with attenuated T2* responses to an acute furosemide stimulus. Although this association was not statistically significant, it would likely achieve significance with a larger study size. This finding is intuitive, given that with most classes of medications, higher chronic daily doses reflect altered pharmacokinetics requiring higher acute doses to achieve a desired physiologic response.

Renal tissue oxygenation is at least partly dependent on oxygen delivery via RBF [18,19]. One limitation of many studies evaluating renal oxygenation with BOLD MR is the reliance on either iodinated or gadolinium based contrast administration for determination of RBF. The former would require a separate imaging examination (computed tomography [CT]), possibly introducing different physiological conditions at separate time points. While RBF and perfusion can be determined during one single imaging examination with the administration of gadolinium contrast, this agent is contraindicated in patients with GFR < 30 ml/min/1.73 m^2^ due to the risk of nephrogenic systemic fibrosis [20], thereby limiting the potential application of this diagnostic strategy in individuals with progressed renal dysfunction.

We employed phase contrast techniques to quantify RBF in each renal artery before and after IV furosemide to determine whether RBF influenced O_2_ utilization. In furosemide naïve participants, RBF increased in response to 20 mg IV furosemide suggesting vasodilation which has been reported previously in human studies [21]. In participants chronically administered furosemide, RBF tended to be lower at baseline and did not increase after 20 mg IV furosemide. This may reflect the higher prevalence of diabetes and/or renal insufficiency in these participants. There was no difference in the severity of stenoses between the two groups.

Change in cortical T2* intensity was correlated with RBF measures, while there was no correlation observed in the medulla. Compared to the cortex, the medulla receives much less blood flow relative to its high metabolic workload and functions in near-hypoxic conditions at baseline [22,23]. Therefore, alterations in RBF and subsequently O_2_ delivery should be better tolerated in the cortex due to its abundance of blood flow [24]. Our findings suggest that the increase in tissue oxygenation observed in the cortex is likely related to increases in total RBF and O_2_ delivery, at least in those not receiving chronic furosemide therapy. However, we observed increases in medullary tissue oxygenation in response to an acute furosemide stimulus although this response was reduced compared to those not receiving chronic furosemide. The relationship between RBF and oxygenation is complex, particularly in the medulla. Increases in RBF do increase renal O_2_ delivery; however, increases in RBF also increase tubular Na + delivery and transport resulting in increased metabolic workload [25]. Our observations may reflect compensatory mechanisms in the medulla to maintain homeostasis and potentially downregulate non-essential metabolic functions in times of reduced RBF and O_2_ delivery that may occur with renal arterial stenoses. Alternatively, RBF may be shunted towards the medulla during stress conditions to maintain metabolic demands as the cortex is relatively overperfused and is likely able to better tolerate reductions in total RBF compared to the medulla. Hence, there is no significant correlation between total RBF and renal medullary oxygenation unless RBF is reduced below a critical point which may be observed in severe renal arterial narrowings.

Our study has some limitations. We were unable to obtain measurements of renal oxygenation by BOLD in 6 of the 76 kidneys due to artifacts (motion or bowel gas) and poor image quality. RBF measures were not performed in 12 of the 76 renal arteries due to inadequate visualization of the renal artery on phase contrast velocity maps due to extremely low blood flow or motion artifact. Second, Na + intake or reabsorption and water loading conditions were not monitored in our study and could potentially confound our results. Another potential confounder is the higher prevalence of participants on ACE inhibitors or ARBs in the furosemide naïve group. We attempted to adjust for these factors in our multivariable analyses. Although these agents theoretically may influence renal tissue oxygenation [26], they did not increase renal issue oxygenation in type 2 diabetic hypertensive patients in a recent study using BOLD MR imaging with a furosemide stimulus [3]. Because the GFRs were different between our two groups, we performed additional analyses with exclusion of five of the furosemide naïve group with GFR >90 to try to minimize some of the between group differences which and the same variables (ethnicity, stenosis severity and chronic daily furosemide dose) remained as significant independent predictors of changes in T2* intensity in response to an acute furosemide stimulus. Our findings are consistent with findings published in a study of 280 patients demonstrating no differences in T2* intensities between different stages of CKD [27]. However, our findings build upon this work by demonstrating no relationship of GFR to changes in T2* signal intensities in response to an acute furosemide stimulus. Because many of these factors are closely linked, future studies specifically aimed at determining the effects of chronic furosemide therapy on acute T2* responses to a furosemide stimulus should be evaluated in better matched cohorts, possibly with differing doses of furosemide stimuli. It would be reasonable to investigate administering a larger intravenous dose of furosemide comparable to the chronic furosemide dose that patients’ receive chronically to be able to detect an appropriate increase in renal oxygenation before determining if a kidney downstream from a severe renal artery stenosis would benefit from a revascularization procedure.

## Conclusions

Our study demonstrates that chronic furosemide therapy attenuates BOLD MR responses to an acute furosemide stimulus in patients with RAS being evaluated for renal artery revascularization procedures. If BOLD MR with a furosemide stimulus becomes a more widely used method for determining clinical outcomes for these procedures, careful attention should be paid to evaluating results in those receiving chronic loop diuretic therapy. Our findings suggest that patients on chronic loop diuretic therapy may require different doses of IV furosemide as a stimulus to detect changes in renal tissue oxygenation and blood flow before BOLD MR is widely used to determine renal viability.

## Competing interests

This study was funded in part by NIH grant R42 AG030248, a Small Business Initiative Grant award to Prova, Inc for which Dr. Hundley and Dr. Hamilton are minor stock holders. All other authors had no disclosures to report.

## Authors’ contributions

MH was involved in scan data acquisition, image analysis, data/statistical analyses, interpretation of data, and drafting the manuscript. MR was involved in study design, interpretation of data, and editing the manuscript. TM was involved in study design, statistical analyses, and editing the manuscript. CH was involved in study design, image acquisition and analyses, interpretation of data and editing the manuscript. ME was involved with study design, patient recruitment, interpretation of data, and editing the manuscript. JJ was involved with image analysis, interpretation of data, and editing the manuscript. JH was involved with patient recruitment and editing the manuscript. WGH was involved with study design, image acquisition and analysis/interpretation, and editing the manuscript. All authors read and approved the final manuscript.

## Authors’ information

WGH is the senior and corresponding author of this manuscript.
